# Structural and Dynamics Insights into Melatonin Binding to MT1 Receptor

**DOI:** 10.1002/cbic.70376

**Published:** 2026-05-15

**Authors:** Clementina Acconcia, Antonella Paladino, Francesca Scebba, Maria della Valle, Martina Montebuglio, Gaetano Malgieri, Carla Isernia, Roberto Fattorusso, Debora Angeloni, Stefano Comai, Luigi Russo

**Affiliations:** ^1^ Department of Environmental Biological and Pharmaceutical Sciences and Technologies University of Campania “L. Vanvitelli” Caserta Italy; ^2^ Institute of Biostructures and Bioimaging, CNR Naples Italy; ^3^ Health Science Interdisciplinary Center Scuola Superiore Sant’Anna Pisa Italy; ^4^ Institute of Crystallography, CNR Caserta Italy; ^5^ The Institute of Biorobotics Scuola Superiore Sant’Anna Pisa Italy; ^6^ Department of Pharmaceutical and Pharmacological Sciences University of Padua Padua Italy; ^7^ Department of Biomedical Sciences University of Padua Padua Italy; ^8^ Department of Psychiatry McGill University Montreal Canada; ^9^ IRCCS San Raffaele Scientific Institute Milan Italy

**Keywords:** melatonin, molecular dynamics simulation, MT1‐receptor, on‐cell membrane NMR spectroscopy

## Abstract

Melatonin (MLT) (N‐acetyl‐5‐methoxytryptamine), mainly acting through its two receptors MT1 and MT2, plays a crucial role in regulating circadian rhythms. In addition, MLT has antidepressant, anti‐inflammatory, anti‐tumor, antioxidant, locomotor activity‐regulating, vascular, and neuroprotective effects. Despite recent reports on X‐ray free‐electron laser and cryo‐EM structures of MT1 receptor in complex with agonists, such as agomelatine, 2‐phenylmelatonin and ramelteon, a detailed high‐resolution description of the MT1 binding by melatonin under near to native (physiological) conditions is still missing. To this aim, we used isolated cellular membranes overexpressing MT1 receptors to conduct a high‐resolution nuclear magnetic resonance interaction study. Combined with computational methodologies such as homology modeling, molecular docking and molecular dynamics simulations, this approach provides significant structural and dynamical insights into the interaction between MT1 receptor and melatonin. We found that MLT interacts, through hydrophobic interactions, with the orthosteric binding pocket enhancing receptor motions that in turn facilitate local rearrangements in the cytoplasmic portion of MT1.

## Graphical abstract

In this study, we describe the structural and dynamic determinants governing MT1 binding by melatonin (MLT) by using an integrated approach that relies on the combination of on‐cell membrane nuclear magnetic resonance structural data with computational techniques. This study demonstrates that MLT, through the indole unit and the methoxy group, binds the orthosteric binding site of the MT1 receptor. The latter, upon binding to MLT, undergoes helical rearrangements.

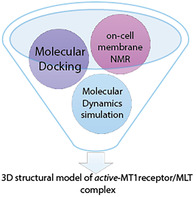

## 1 Introduction

Melatonin (MLT) is a hormone synthetized from serotonin by the pineal gland during the night [1, 2]. Its synthesis is strongly modulated by the suprachiasmatic nucleus of the hypothalamus, which transmits photoinformation received from the retina to the pineal gland by a multisynaptic pathway [3]. MLT plays a regulatory role in different physiological and behavioral processes such as circadian rhythms, sleep–wake cycle, thermoregulation, seasonal adaption, hormonal secretion, reproduction, and digestion [4, 5]. Additionally, MLT when administered at pharmacological doses has shown anti‐inflammatory, antioxidant, analgesic, hypnotic and neuroprotective effects [2, 6, 7, 8, 9, 10, 11, 12, 13]. In addition to antioxidant and free radical scavenging properties due to its chemical structure, most of the MLT actions in the body arise from its interaction with two high affinity G protein‐coupled receptors (GPCRs) namely melatonin receptor‐1 (MT1) and melatonin receptor‐2 (MT2). Although MT1 and MT2 receptors are implicated in different biological processes and pathologies of the brain and the periphery [13, 14, 15, 16, 17, 18, 19], they share the canonical structural architecture of the family A GPCRs [20, 21]. Recently, the 3D structures of the *inactive* MT1 and MT2 receptors in complex with agonists (i.e., agomelatine (MT1 PDB 6me5), 2‐phenylmelatonin (MT1 PDB 6me3; MT2 PDB 6me6), ramelteon (MT1 PDB 6me2; MT2 PDB 6me9), and 2‐iodomelatonin (MT1 6me4) were solved by X‐ray free‐electron laser (XFEL) technique [20, 21]. Although both MLT receptors were crystallized in an inactive conformation, XFEL data provided valuable high‐resolution details of the structural determinants governing the recognition mechanisms of ligands by the receptors. In particular, XFEL 3D structures showed that MT1 and MT2 receptors present a similar fold consisting of a transmembrane (TM) heptahelical (TM1‐TM7 helices) bundle, a short amphiphilic helix (helix 8) that is parallel to the cytoplasmatic surface of the membrane, three extracellular loops (ECLs) and three intracellular loops (ICLs). A structural hallmark of MT1 and MT2 receptors is a high level of primary sequence conservation in the binding pocket [22]. Yet, the comparison of the XFEL structures demonstrated that both receptors present a lateral channel between TM4 and TM5, allowing ligand access to the orthosteric site, that is slightly wider and more evident in MT1 with respect to MT2. More recently, the cryo‐EM structure of the MT1‐Gi signaling complex revealed the molecular basis of the agonist‐bound activation mechanism of MT1 receptor [23]. In particular, cryo‐EM data demonstrated that the MT1 activation by ramelteon occurs through a large outward movement of TM6 allowing the transmission of signals from the ligand pocket to the cytoplasmatic side of the receptor. In a recent research, we have shown that the selective MT1 receptor partial agonist UCM871 was able to reverse the manic‐like phenotype of Clock mutant mice by promoting the shift from the inactive to the active state of MT1 receptors [24]. In particular, we demonstrated that the binding of UCM871to the orthosteric pocket of the MT1 receptor, mainly by hydrophobic interactions, activates the receptor via a molecular mechanism driven primarily by conformational rearrangements on the cytoplasmic side of the receptor.

In this study, we aim to elucidate the structural and dynamic determinants governing MT1 binding by MLT, that in turn regulate the activation of the receptor, through a multidisciplinary approach combining NMR structural data with computational techniques (Figure S1). First, we conducted NMR binding studies of MLT to MT1 receptors using isolated cell membranes overexpressing the receptor. Second, a computational analysis supported by NMR data provided a 3D model of the active MT1 receptor bound to MLT, revealing the details of the receptor‐ligand interactions. Third, to understand the effect of MLT binding on MT1 dynamics, we performed molecular dynamics (MD) simulation studies of the receptor in both inactive and active‐MLT bound forms in fully solvated and membrane‐embedded states. Our results demonstrate that MLT occupies the orthosteric binding site of MT1, primarily forming hydrophobic contacts with residues located within ECL2 and localized at TM3, TM4, TM5, and TM7 α‐helices. Furthermore, we show that MLT binding enhances the structural dynamics of MT1, facilitating local rearrangements in the cytoplasmic part of the receptor involved in the activation mechanism.

## 2 Results and Discussion

### 2.1 NMR Investigation of MLT‐MT1 Interaction

**FIGURE 1:**
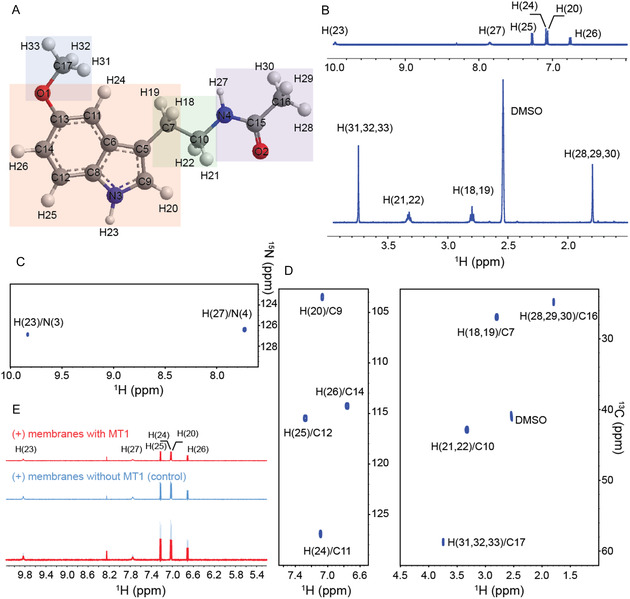
NMR investigation of MLT in the absence and presence of MT1 cell membranes. (A) Chemical structure of MLT in which the numerical labeling of atoms used throughout the manuscript is reported. In the panel A, the four subunits of the molecule highlighted are methoxy group (light blue), indole unit (light red), methylene unit (light green) and amide group (light purple). (B) Extended sections of 1D ^1^H NMR spectrum of melatonin acquired on 600 MHz spectrometer at 298 K. (C) A portion of the ^1^H‐^15^N HSQC spectrum of MLT. (D) Expansion of aromatic (left) and aliphatic (right) regions of the ^1^H‐^13^C HSQC spectrum of melatonin. (E) Aromatic region of 1D ^1^H spectra of MLT acquired in the presence of cell membranes with (red) and without overexpressed MT_1_ receptor (light blue). This latter experiment was used as control in order to exclude nonspecific interactions. The overlay of 1D ^1^H spectra is also reported. In all NMR spectra the resonances assignment is reported.

To provide a rigorous NMR investigation of the molecular determinants driving the MLT‐MT1 receptor interaction, an accurate assignment of detectable ligand‐free resonances is required. Therefore, we first investigated MLT in the free form by natural‐abundance NMR spectroscopy (Figure 1A–D). A nearly complete assignment of ^1^H, ^13^C, and ^15^N chemical shifts at 298 K (Figure S2) has been obtained by simultaneously analyzing heteronuclear experiments such as 2D[^1^H‐^13^C] HSQC and 2D [^1^H‐^15^N] HSQC with 2D [^1^H‐^1^H] TOCSY, 2D [^1^H‐^1^H] ROESY, and 2D [^1^H‐^1^H] NOESY spectra (Figure S3A). The ^1^H NMR spectrum of free‐MLT (Figure 1B) shows two signals at 9.83 and 7.77 ppm corresponding to exchangeable H_N_ protons H23 and H27, respectively. In the region of 6.77–7.28 ppm the signals corresponding to three protons of the indole unit (i.e., δ H(20) = 7.07 ppm, δ H(24) = 7.09 ppm, δ H(25) = 7.28 ppm, and δ H(26) = 6.77 ppm) were found. The singlet integrating for three protons (H(31,32,33)) of the methoxy group resonates at 3.74 ppm. The signals corresponding to H(18,19) and H(21,22) protons of the methylene unit were observed in the spectrum at 2.80 and 3.33 ppm, respectively. The protons of the acetyl CH_3_ (H(28), H(29) and H(30)) within the amide group were detected as singlet at 1.76 ppm. As mentioned above, free‐MLT was also characterized by natural‐abundance 2D heteronuclear experiments (Figure 1C,D). In particular, in the aromatic 2D [^1^H‐^13^C] HSQC spectrum (Figure 1D) the indole unit protons H(20), H(24), H(25), and H(26) were observed to correlate with the aromatic carbons C(9) (*δ* = 103.39 ppm), C11 (*δ* = 126.90 ppm), C12 (*δ* = 115.47 ppm) and C14 (*δ* = 114.26 ppm), respectively. In addition, the inspection of the aliphatic 2D [^1^H‐^13^C] HSQC spectrum (Figure 1D) allowed us to assign carbons of the methoxy, methylene and amide units: the singlet for the three methoxy protons correlated with the C17 carbon signal at *δ* = 58.72 ppm; the proton signals of the methylene unit H(18,19) and H(21,22) correlated with the C7 (*δ* = 26.92 ppm) and C10 (*δ* = 42.85 ppm) carbons, respectively; the singlet of the acetyl CH_3_ was observed to correlate with C16 (*δ* = 24.80 ppm). Yet, the chemical shifts assignment of protonated nitrogen atoms of MLT was performed by analyzing the 2D [^1^H‐^15^N] HSQC (Figure 1C). In this latter spectrum the amide protons H(23) and H(27) correlated with N(3) (*δ* = 126.88 ppm) ppm and N(4) (*δ* = 126.40 ppm), respectively. After that, to get insight into the molecular determinants governing the MT1 binding by MLT, we monitored the MT1‐ligand binding by NMR spectroscopy. First, we acquired 1D and 2D ^1^H NMR spectra of MLT in the presence of isolated cell membranes overexpressing the MT1 receptor (Figure 1E and S3B, C). Upon addition of membranes containing the MT1 receptor (MT1‐membranes), a clear decrease in relative ^1^H signal intensity without migration of peaks or appearance of MT1‐bound state resonances was observed for all MLT protons with those located within the indole unit and the methoxy group showing a significant line broadening. These observations, in line with previously reported K_D_ value [25], indicate that MT1/MLT complex exists in a slow/slow‐intermediate exchange on the NMR timescale. To quantify the reduction of ^1^H MLT signals due to the interaction with MT1, we calculated the relative attenuation factor AF (%) (for details see materials and methods) for each proton of the ligand by comparing the individual signal intensities in the ^1^H NMR spectra of MLT acquired upon addition of cell membranes with and without the MT1 receptors. This latter condition allowed us to remove from the analysis the effect of possible nonspecific interactions with different cellular membrane components. As reported in Figure S4, AF values suggest that MLT interacts with the MT1 receptor through a binding mechanism which is mainly driven by the indole unit and the methoxy group with the methylene unit that contributes to stabilize the MT1/MLT complex. However, to integrate the NMR data analysis providing a clearer description of the epitope map of MLT to MT1, we further investigated the MT1‐MLT interaction by using a series of computational techniques such as molecular modeling, molecular docking and MD simulations.

### 2.2 Structure and Dynamics of the Inactive‐Unbound MT1 Receptor

**FIGURE 2:**
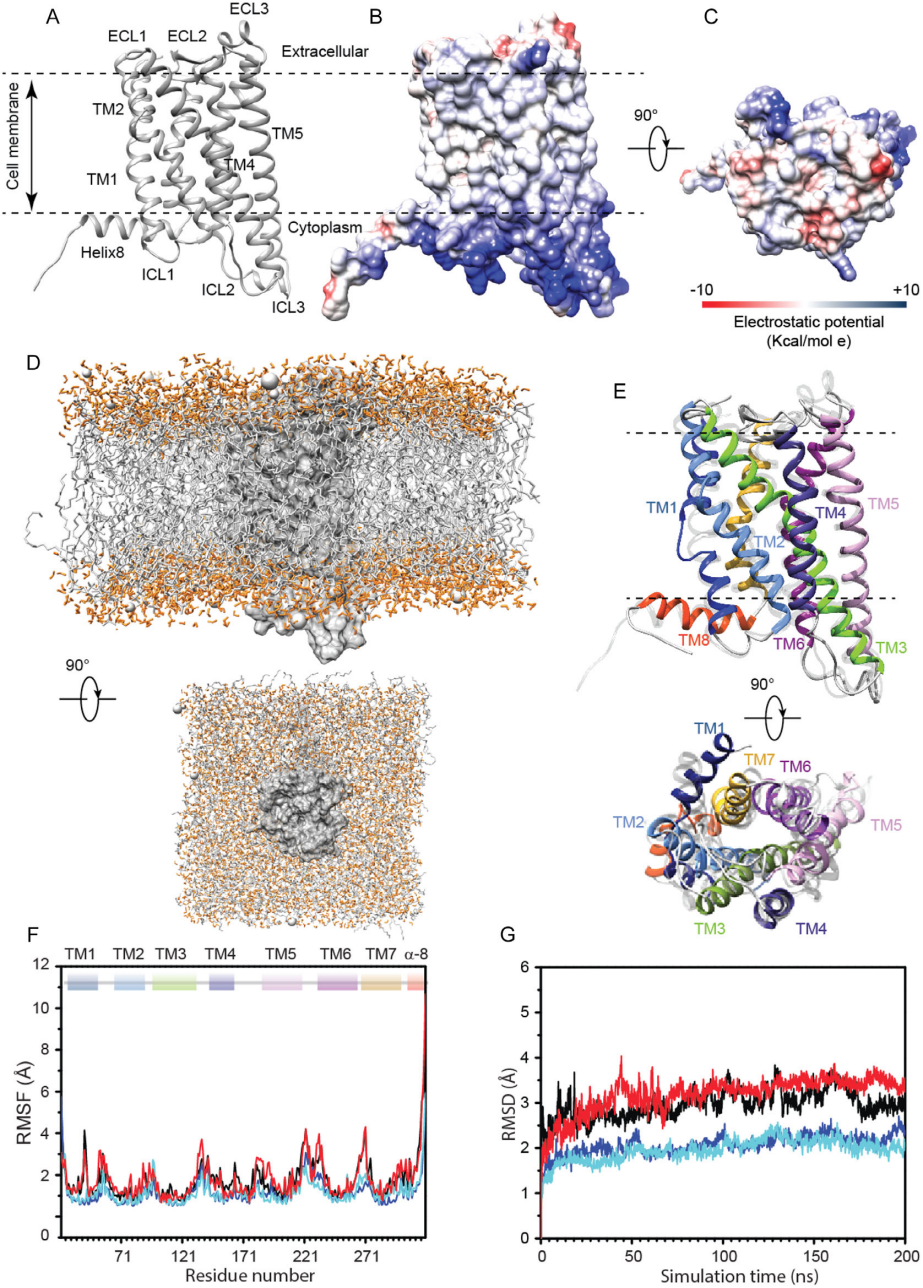
Structure and dynamics of the *inactive*‐unbound MT1 receptor. (A–C) Inactive state of MT1. (A) Ribbon drawing representation of the *inactive*‐unbound MT1 receptor. The seven TM helices, the connecting ICLs and ECLs loops are indicated. The short helix (α8) that lines parallel to the cytoplasmic face of the membrane is also reported. (B,C) Electrostatic surface potential map of the *inactive*‐unbound MT1 receptor. The surface is depicted from electropositive (blue; 10 kcal/mol) to electronegative (red; −10 kcal/mol). (D–G) MD simulations results. (D) Starting structure of the membrane‐embedded inactive state MT1, only a reduced fraction of the solvation box is displayed for clarity. (E) Ribbon drawing diagram of the representative structure of the most populated cluster from the MD simulation of the *inactive*‐unbound MT1 receptor in two orientations rotated 90° with respect to the *x* axis. The TM helices are depicted using the following color code: TM1 (blue), TM2 (cornflower blue), TM3 (light green), TM4 (dark slate blue), TM5 (plum), TM6 (dark magenta), TM7 (goldenrod), Helix8 (α8) (orange red). (F), (G) RMSF and RMSD plots of Cα atoms for the *inactive*‐MT1 receptor in absence of the ligand are reported in the presence and absence of the membrane model for the two independent replicas (R1/R2 in solvent membrane black/red; R1/R2 in membrane blue/cyan).

First, we explored the structural and dynamical peculiarities of the unbound MT1 receptor in its inactive state. The structural model of the *inactive*‐unbound MT1 receptor, comprising the region from Arg^22^ to Phe^320^, was built by adding missing regions to the partially‐resolved X‐ray structure of the receptor in complex with ramelteon, as reported in materials and methods (Figures 2 and S5). To note, the Ramachandran plot analysis indicates an excellent quality of the predicted structural model with 100% residues lying in energetically allowed regions for the (φ, ψ) torsion angles (most favored and additional allowed regions) (Figure S5A). As reported in Figure 2A, the 3D model of the *inactive*‐unbound MT1 exhibits the expected architecture of GPCRs with seven TM helices (TM1 = Ser^24^‐Arg^54^; TM2 = Ile^64^‐Asn^90^; TM3 = Tyr^97^‐Lys^134^; TM4 = Ser^144^‐Ala^165^; TM5 = Ser^185^‐Val^217^; TM6 = Arg^235^‐Ser^263^; TM7 = Glu^274^‐Leu^298^) followed by a short α‐helix (α‐8 = Gln^300^‐Ser^312^) that reclines parallel to the cytoplasmic‐side of the cell membrane. The 7TM helices are connected by three intracellular loops (ICL1= Asn^55^‐Asn^63^; ICL2=Tyr^135^‐Asn^143^; ICL3=Arg^218^‐Phe^234^) and three extracellular loops (ECL1= Asn^91^‐Gly^96^; ECL2= Gly^166^‐Ser^184^; ECL3= Asp^264^‐Pro^273^). Yet, The electrostatic surface potential analysis indicated that the cytoplasmic portion of the MT1 receptor is rich of positively charged residues; on the contrary, outside the cell, the receptor presents mainly regions lacking polarity of the atoms with a slightly negatively charged surface (Figure 2B,C). To validate the structural model and gain insights into the dynamics properties of the 3D structural model of the receptor, MD simulations studies were carried out on unbound MT1 in its inactive state (Figure 2D–G). It is worth noting that in addition to the membrane‐embedded systems, MT1 receptor was simulated out of the membrane to enhance the assessment of local fluctuations. This approach is also employed to evaluate potential biases in the predicted models which might arise from force field accuracies.

MD studies showed significant structural stability of the *inactive*‐unbound MT1 receptor during the entire simulation time. The TMs helical assembly samples minimal structural adjustments with root mean square deviation (RMSD) Cα (Figure 2G) values <3 Å on average: indeed, the large intracellular‐loop between TM5 and TM6 (ICL3) represents the most fluctuating motif within the receptor while TM4‐TM5 β‐hairpin (ECL2) was endowed with small variability. Root mean square fluctuations (RMSF) also highlighted a spike region within the TM1, corresponding to the kink of the helix (Figure 2E,F) and amino acid residues Asp^41^, Ile^42^, and Leu^43^. This overall stability was also confirmed by the large conservation of the secondary structures content during the simulation time (Figure S6).

### 2.3 Structural Dynamics of the Active‐MT1 Bound to MLT

**FIGURE 3:**
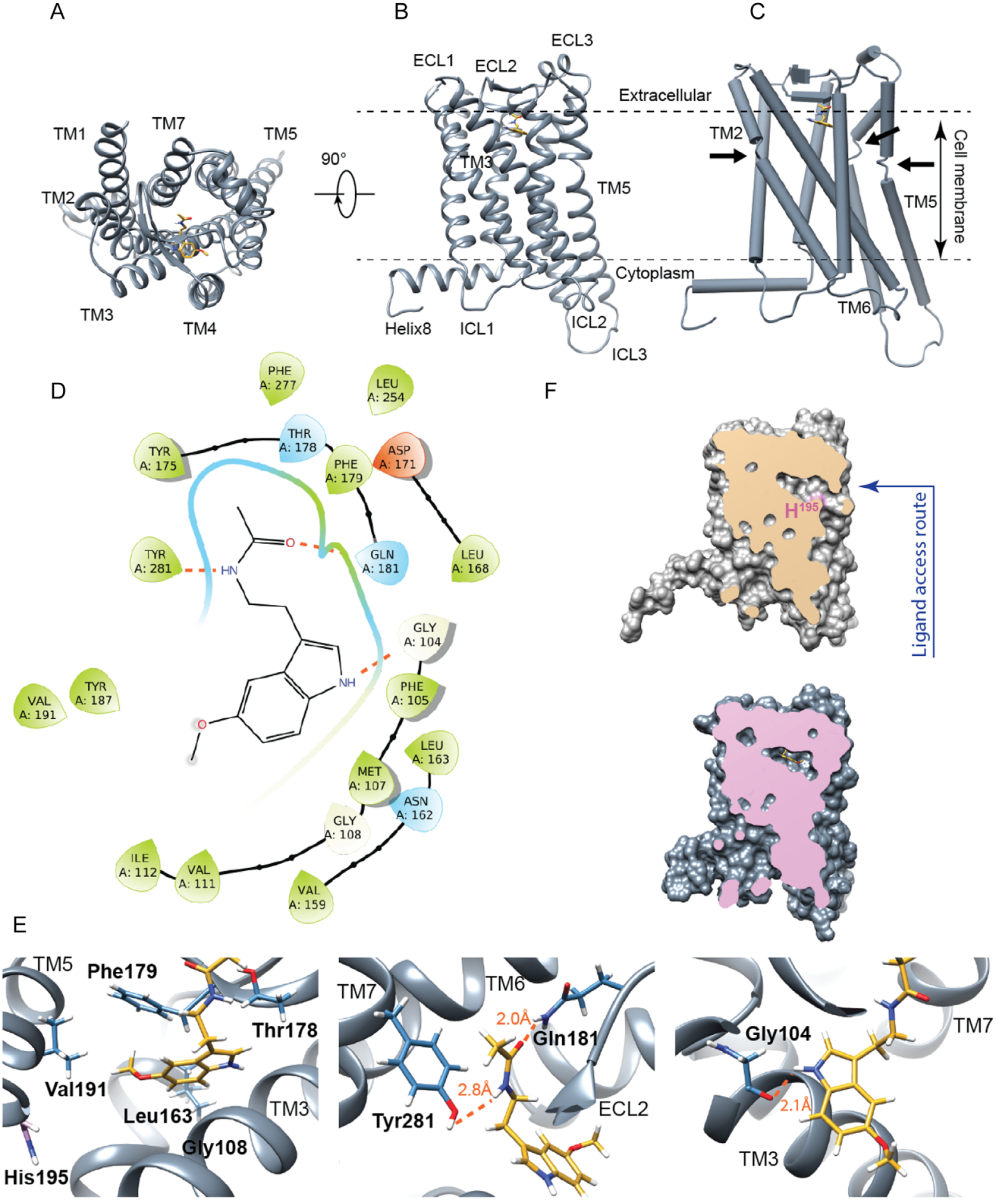
Structural details driving the recognition mechanism of the active state of MT1 receptor by MLT. (A), (B) The *active*‐MT1/MLT complex, obtained using computational data, in two orientations (top (A) and front (B) view) rotated of 90° around *x* axis. The *active*‐MT1 receptor is reported as slate gray ribbon diagram whereas MLT is illustrated as yellow stick model. (C) Structural features of the *active*‐MT1 receptor in complex with MLT. The kinks in TM2, TM5, and TM6 caused by interruptions in the intra‐helical hydrogen bonding are highlighted. (D) 2D interaction scheme of the MT1 residues forming the MLT binding surface. The orange dashed lines denote hydrogen bonds crucial in the complex stabilization. Negative charged, polar and hydrophobic residues are painted in orange, light blue and light green, respectively. (E) Close‐up views of the *active*‐MT1/MLT complex. The MT1 residues playing a crucial role in the MLT binding are highlighted. The side chains of the *active*‐MT1 receptor directly involved in the interaction are illustrated as light blue stick model; whereas the side‐chain of the highly conserved His^195^ residue is depicted as light pink. MLT is reported as yellow stick. (F) (upper) Ligand access route of the MT1 receptor in which is highlighted the highly conserved His^195^ residue. (lower) Surface representation of the *active*‐MT1 receptor in complex with MLT in which the orthosteric binding site is highlighted.

To provide an accurate description of the MT1‐MLT interaction, we applied a multidisciplinary approach in which computational techniques such as homology modeling, molecular docking and MD simulations were compared to experimental NMR‐based structural data (Figure S1). First, the 3D structural model of the MT1 receptor in the active state was built using the software MODELLER [26] (for details see materials and methods) and its quality was assessed by PROCHECK through Ramachandran plot analysis (more than 99% residues in most favored and additional/generously regions) (Supporting Information Figure S5B); successively, the MLT was docked on the obtained 3D structure of the *active*‐MT1 by AutoDock Vina program [27] (for details see materials and methods). As a results, the molecular docking calculation generated 100 conformers that were sorted into clusters based on the binding energy values. This procedure produced three different clusters, characterized by different binding energy and showing small differences, in terms of ligand orientation relative to the binding site, in the molecular recognition of MT1 receptor by MLT. Hence, we selected as representative model of the *active*‐MT1/MLT complex the lowest‐energy structure of the most populated cluster 1 (44%). In good agreement with the NMR data, the 3D model of the *active*‐MT1/MLT complex (Figure 3A–F), indicates that MLT binds the orthosteric binding site of the receptor with specific contacts to ECL2 and TM3, TM4, TM5, and TM7 α‐helices (Figure 3D,E). In details, the amide proton of the indole unit (H(23)) of the MLT is hydrogen bonded with the backbone carbonyl of the Gly^104^ (TM3), whereas the H(27) proton and the oxygen (O2) of the amide group form hydrogen bonds with the Tyr^281^(TM7) and Gln^181^(ECL2), respectively. In addition, the *active*‐MT1/MLT complex is strongly stabilized by an extensive network of hydrophobic interactions. Particularly, the methoxy group is deeply embedded into a hydrophobic cleft formed by Leu^163^ (TM4) and Val^191^(TM5); the indole unit forms hydrophobic interactions with Gly^108^(TM3) and Leu^163^ (TM4); the methylene unit makes hydrophobic contacts with Gly^108^(TM3) and Phe^179^(ELC2); the acetyl CH_3_ of the amide group fit into a polar groove composed by Thr^178^(ELC2), Gln^181^(ELC2), Tyr^281^(TM7). To better understand the structural basis underlying the mechanisms by which ligands with variable efficacies differently modulate the conformational changes of the receptor, we compared the obtained structural model of the *active*‐MT1/MLT complex with the Cryo‐EM structure of the active‐state MT1 bound to the ramelteon [20]. Interestingly, as reported in Figure S7, MLT and ramelteon bound to the orthosteric site of the *active*‐MT1 receptor show slightly different binding poses that are stabilized by a specific set of interactions in the ligand‐binding pocket. In particular, the key structural difference between MLT and ramelteon is that the former makes several contacts with residues located within the TM7, whereas the latter forms hydrophobic interactions with residues of TM6 helix. Overall, our findings show that MLT and its analog present a slightly dissimilar binding interface into the orthostatic pocket of MT1 indicating that, most likely, the two ligands are characterized by a lightly different activation mechanism. MLT reaches the *active*‐MT1 orthosteric binding pocket by a lateral ligand channel containing many hydrophobic residues and the highly conserved His^195^. Regarding the role of the histidine residue (His^195^) in the MT1 binding mechanism, mutagenesis studies reported by Laitinen and coworkers [28] showed that the substitution of His^195^ with alanine reduces the MT1 receptor affinity for 2‐[^125^I] iodomelatonin. Although a minor reduction of the binding affinity was observed, the authors hypothesized a direct involvement of the histidine in the binding of ligands by MT1. In contrast, XFEL structures of the *inactive*‐MT1 in complex with agonists [21] (Figure S8), as well as the cryo‐EM structure of the *active*‐MT1 bound to ramelteon [23] (Figure S7), revealed that His^195^ does not interact with ligands. These findings are fully supported by the obtained *active*‐MT1/MLT structural model indicating that His^195^ is not directly involved in the binding of MLT (Figure 3F). Therefore, the 3D model of the *active*‐MT1 receptor in complex with MLT, together with previously published structural and mutagenesis data [28], demonstrates that the histidine residue (His^195^) plays an indirect role in the binding mechanism of MLT and its analogs. Yet, the comparison between the 3D structural model of the *active* MLT‐bound MT1 with the *inactive* form reveals that upon binding to MLT, the regions of the receptor close to the cytoplasmic side undergo significant structural rearrangements (Figure S9). Notably, the structural deviation between the two MT1 states changes over time with the Cα RMSD increasing from 1.28 Å, in the starting structure, to 4.30 Å and the end of the simulation. Accordingly, TM5 and TM6 showed a comparable increasing structural displacement of around 1 Å along the MD trajectory (Table S1).

**FIGURE 4:**
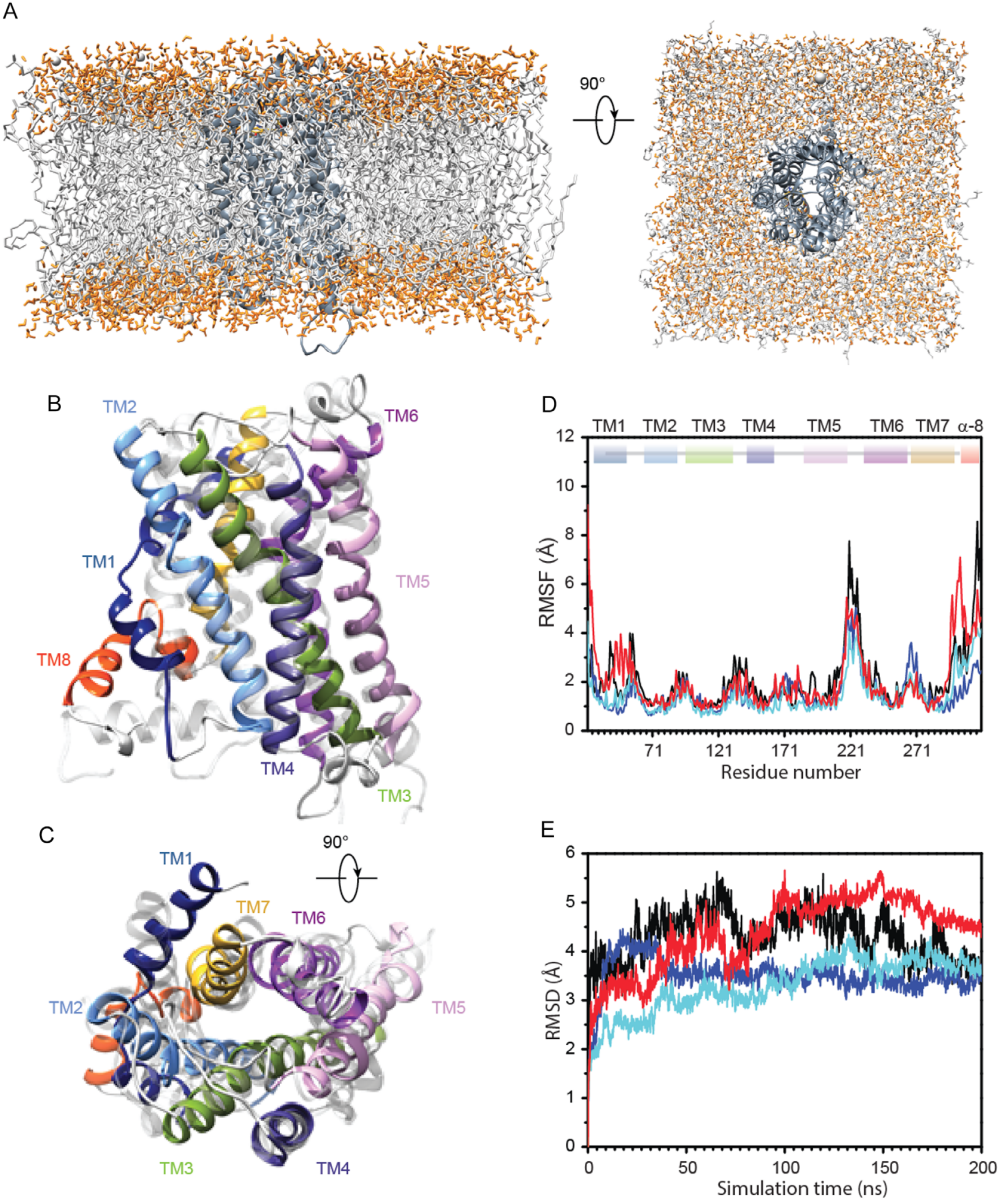
Molecular dynamics simulation analysis of the *active*‐MT1 receptor in complex with the melatonin. (A) Starting structure of the membrane‐embedded MLT/*active*‐MT1 complex, only a reduced fraction of the solvation box is displayed for clarity. (B), (C) Ribbon drawing diagram of the representative structure of the most populated cluster from the MD simulation of *active*‐MT1 receptor in complex with MLT in two orientations rotated 90° with respect to the *x* axis. The TM helices are depicted using the following color code: TM1 (blue), TM2 (cornflower blue), TM3 (light green), TM4 (dark slate blue), TM5 (plum), TM6 (dark magenta), TM7 (goldenrod), Helix‐8 (α8) (orange red). (D), (E) RMSF and RMSD plots of Cα atoms for the *active*‐MT1/MLT system in solvent and membrane for the two independent replicas (R1/R2 in solvent membrane black/red; R1/R2 in membrane blue/cyan).

To further validate the predicted interaction model, we set up MD simulations for the *active*‐MT1/MLT complex to gain insights into the stability of the recognition and its effects on the dynamical accommodation of the receptor, in both fully solvated and membrane‐embedded states (Figure 4A–E). Comparative analyses disclose enhanced structural deviations of MT1 upon MLT binding (Figure 4A–E), with respect to the inactive MT1 state. This observation is also confirmed by the cluster analysis showing that the most populated cluster (cluster MT1‐MLT #1) only represents ∼30% of the entire conformational ensemble (note that cluster *inactive*‐unbound MT1 #1 ∼ 44%). Enhanced conformational dynamics of the MT1 receptor upon binding is also visible from principal component analysis (PCA) analysis carried out on both systems and shown in Figure S10 (see Materials and Methods section).

Moreover, time‐averaged RMSF per residue highlights increased flexibility at the TM5‐TM6 connecting loop (ICL3) (Figure 4D). The structural superposition of the two complexes (i.e., first cluster structure *per* system) shows marked structural adjustments and reciprocal motions among some of the TM helices proven to concur to the MT1 activation process (Figure S11A, B). In the starting *active*‐MT1/MLT complex, MLT is stabilized by several interactions at the receptor binding site, establishing electrostatic and hydrophobic contacts with TM helices and the ECL2 motif as well. MD data confirm the stability of the recognition, showing that the interaction network is well conserved along the simulation time and several amino acid residues from TM3, TM4, TM5, TM6, and TM7 stabilize MLT binding pose (Figure 3D). In line with these findings, mutation of key interacting residues results in an average change in free energy (ΔΔG) of 1.47 kcal/mol for Q181A and 2.22 kcal/mol for Y281A mutants relative to the *wild‐type*, indicating a significant destabilization of the complex (Table S2). These results support a central role for these residues in the MT1‐MLT interaction.

Furthermore, we demonstrate that MLT binding modulates the structural flexibility of the MT1 receptor: cluster analysis on the concatenated trajectories of *inactive*‐unbound MT1 and *active*‐MT1/MLT shows that the presence of the ligand enhances the structural dynamics of the protein. Structural comparison between the two clusters centroids show that most of the flexibility is recorded at the TM5–TM6 connecting loop, at the cytoplasmic side (Figures 4 and S11B). Interestingly, previous studies demonstrated significant helical rearrangements during the activation mechanism of several GPCRs [29]. Among these, a peculiar TM6 outward displacement coupled with a sliding motion has been reported for MT1 in the active conformation [30]. Therefore, we follow some characteristic inter‐residues distances (namely CαN^143^TM4‐CαN^236^TM6) in the two systems along the simulation time as indicative of MT1 activation state [21]. We observe that the initial distance differences in the *active*‐MT1/MLT bound state (∼24.4 Å) and the inactive state (∼20.7 Å) are kept during the entire MD trajectory (data not shown).

To investigate eventual structural rearrangements upon binding, global intra‐helices motions have been evaluated by analyzing reciprocal rotations and distances. TM6 is reported as the main structural discriminant, endowed with the largest fluctuation. We define all the helical axes that correspond to TM1−7 and calculate mutual rotations and distances in both systems. We compare the distribution of inter‐helical distances with respect to TM6, omitting peripheral flexible elements TM1 and α8. In this framework and considering the limited structural changes imposed by membrane restraints, we focus on fully‐solvated receptors simulations to gain insights into local adjustments that might indicate potential major accommodations. Figure S12 shows distance distributions between the geometric center of mass of TM helices: inter‐helical distances in the *inactive‐*unbound MT1 system sample very homogeneous values compared to the MLT bound receptor, which is characterized by multimodal distributions for some of the calculated distances. In particular, appreciable helical rearrangements localize at the interface between TM6 and TM2/TM3/TM4: the average value of the TM2–TM6 distance is 18.47 ± 0.54 Å in the *inactive*‐unbound MT1 becoming 20.13 ± 1.36 Å in the active‐MT1/MLT system, while for TM4/TM6 is 20.1 ± 0.63 Å and 22.3 ± 0.86 Å, respectively.

It is worth underscoring that we plot inter‐distances between the geometric center of mass of the helix, thus averaging the most visible differences emerging at the terminals of the helices (Figure S11). A very early sliding movement of the TM6 toward the cytoplasmic side of the membrane can be also glanced in the initial conformational sampling (Figure S11B).

## 3 Conclusion

In this study, we investigated the molecular determinants driving the MT1 binding by MLT under near to native (physiological) conditions. To decipher the mechanism by which MLT interacts with the receptor, we applied a combined approach integrating high‐resolution structural and dynamical NMR data, collected using cell membranes overexpressing MT1 receptor, with computational techniques such as molecular modeling, molecular docking and MD simulations. First, we investigated the molecular mechanism by which MLT interacts with the MT1 receptor using in‐membrane 1D and 2D ^1^H NMR experiments. NMR data analysis suggested that the formation of the *active*‐MT1/MLT complex is mainly driven by the methoxy group and by the indole ring of the ligand. Second, to obtain a 3D structural model of the *active*‐MT1/MLT complex we performed a series of molecular docking calculations. These latter studies indicated that the ligand is accommodated into the orthosteric binding pocket of the receptor, making specific contacts to ECL2 and TM3, TM4, TM5, and TM7 α‐helices. Interestingly, MLT shows a MT1 binding site slightly different from those observed for other agonists (i.e., agomelatine, 2‐phenylmelatonin, and ramelteon), forming a distinct network of contacts that affect the relative orientation of MLT into the orthosteric pocket. Third, to understand the conformational changes of the MT1 architecture induced by MLT, we compared the structural and dynamical characteristics of the *inactive*‐MT1 with those observed for the *active* form of the receptor in complex with the MLT. Taken together, NMR and MD simulations data indicate that upon binding to MLT the MT1 receptor undergoes helical rearrangements localized at the interface between TM6 and TM2, TM3, and TM4 helices. Specifically, upon interaction to MLT, TM6 shows an average structural displacement of 3.2 and 2.7 Å outward the TM2 and TM4, respectively. This advancement in the field has significant implications for drug development, particularly for the design and synthesis of melatonergic drugs that are selective for MT1 receptors. Indeed, melatonin MT1 receptors have been shown to be potentially implicated in the neurobiology and/or psychopharmacology of bipolar disorders [24], rapid eye movement (REM) sleep [19], Parkinson's disease [31], ischemic stroke [32], and Huntington's disease [33]. By elucidating the specific structural and dynamic changes involved MT1‐MLT interaction, we provide structural insights useful for the rational design of new therapeutic compounds. These compounds can be tailored to exploit the unique binding site characteristics and activation mechanisms of MT1, thus enhancing specificity and efficacy, and likely leading to fewer off‐target effects and improved therapeutic profiles.

## 4 Experimental Section

### 4.1 Cell Culture and Transfection

HEK293T cells were preserved in Dulbecco's modified Eagle's medium Low Glucose supplemented with 10% Fetal Bovine Serum (FBS, Gibco), 200 U/ml Penicillin/Streptomycin, 4 mM L‐Glutamine. By polyethylenimine (PEI) in the 1:3 DNA/transfectant ratio, cells were transfected with the Homo sapiens melatonin receptor 1A cDNA (MTNR1A, NM_005958), into pEZ‐M07 EX‐A0757‐M07 plasmid, featuring 3XHA tag (Gene Copoeia, Rockville, MD USA). After 72 h, cells were fixed for immunofluorescence with anti‐HA antibody, to check for transfection efficiency, or collected for additional analysis. All reagents were purchased from Sigma (St. Louis, MO) unless differently indicated.

### 4.2 Protein Extraction and Western Blot

Cell pellets were prepared with Radio‐Immuno Precipitation Assay lysis buffer (50 mM Tris HCl, 150 mM NaCl, 1.0% v/v NP‐40, 0.5% w/v Sodium Deoxycholate, 1.0 mM EDTA, 0.1% w/v SDS, 0.01% w/v sodium azide at pH 7.4) added of proteases and phosphatases inhibitors. Successively, MNTR1A protein was immunoprecipitated with anti‐HA beads agarose (Pierce) and separated from beads with the soft elution method using HA peptide, followed by dialysis against 1X phosphate‐buffered saline (PBS). The obtained protein from immunoprecipitation was analyzed by Western Blot and tested positive using either anti‐HA antibody or anti‐MTNR1A antibody, not shown here.

### 4.3 Membrane Preparation

To get the cell membrane fraction, in according to Abcam protocol, HEK293T cells transfected for MNTR1A overexpression were processed for subcellular fractionation. Briefly, cells were lysed with ice‐cold Fractionation Buffer (20 mM Hepes pH 7.4, 10 mM KCl, 2 mM MgCl2, 1 mM EDTA, 1 mM EGTA), 1 mM DTT, and protease inhibitors. The cell lysate was passed through a 27G needle and centrifuged (5424R, Eppendorf, Germany) at 720 g for 5 min, to pellet nuclei. After that, to remove mitochondria, the supernatant was collected and centrifuged again at 10 000 g (5424R, Eppendorf, Germany) for 5 min. Then, the supernatant was collected and centrifuged at 100 000 g (Optima LE80K, Beckmann, USA) for 1 h, the pellet was resuspended, passed through a 25G needle and centrifuged again at 100 000 (Optima LE80K, Beckmann, USA) for 45 min. Finally, the pellet was resuspended in 50 µl of 1X PBS. Membranes were prepared either from untransfected or MNTR1A‐overexpressing cells, with a yield in total protein content of about 5–6 µg/µl. 50 µl‐aliquots of both preparation types were used to prepare, as reported in the next paragraphs, NMR samples for the binding experiments.

### 4.4 NMR Measurements

NMR experiments were acquired at 298 K by using a Bruker AVIII HD 600 MHz spectrometer equipped with a triple resonance Prodigy N2 cryoprobe and a *z*‐axis pulse field gradient. The NMR sample was prepared by dissolving MLT in 200 μL of 20 mM sodium phosphate, 0.15 M NaCl (PBS) buffer pH 7.8,10% H_2_O, and 10% DMSO‐d6 in a 3 mm NMR tube for a final concentration of 400 μM.^1^H, ^15^N and ^13^C chemical shift assignments of MLT were obtained by acquiring and analyzing the following NMR spectra: 1D ^1^H spectra were acquired with a spectral width (SW) of 6009.62 Hz, relaxation delay 3.0 s, 128 K data points for acquisition and 256 K for transformation. Bi‐dimensional (2D) [^1^H‐^1^H] Total Correlation SpectroscopY (TOCSY) [34], 2D [^1^H‐^1^H] Nuclear Overhauser Effect SpectroscopY (NOESY) [35] and 2D [^1^H‐^1^H] Rotating frame Overhauser Effect SpectroscopY (ROESY) [36] were acquired using 32 scans for t1 increment, SW of 6009.62 Hz along both t1 and t2, a relaxation delay of 3.0 s and 2048 x 140 complex points in t2 and t1, respectively. The 2D [^1^H‐^1^H] TOCSY spectrum was acquired by a homonuclear Hartman–Hahn transfer using a MLEV17 mixing sequence with a mixing time of 70 ms and a 10 kHz spin‐lock field strength; 2D [^1^H‐^1^H] NOESY experiments were recorded with a mixing time of 250 ms; and 2D [^1^H‐^1^H] ROESY experiments were carried out with a spin‐lock field strength of 4 kHz and a mixing time of 250 ms. In 2D homonuclear NMR experiments, water suppression was achieved by Watergate pulse sequence with application of gradients using double echo scheme. 2D [^1^H‐^1^H] NMR experiments were apodized with a square cosine window function and zero filled to a final matrix of 4096 x 1024 before the Fourier transform and baseline correction. 2D hetero‐nuclear [^1^H‐^15^N] Heteronuclear Single Quantum Coherence Spectroscopy (HSQC) NMR experiment was acquired with 880 scans per each t1 increment, 1.0 s relaxation delay, SW of 1581.26 Hz along t1 and 6009.62 Hz along t2, 2048 x 128 complex points in t2 and t1, respectively. The ^1^H‐^15^N HSQC pulse sequence was optimized, as reported in a previous publication [37], starting from a Bruker preprogrammed gradient echo‐antiecho program [38]. The 2D [^1^H‐^15^N] experiment was apodized with a square cosine window function and zero filled to a final matrix of size 4096 x 1024 before Fourier transform and baseline correction. Aliphatic 2D hetero‐nuclear [^1^H‐^13^C] HSQC spectrum was carried out with 128 scans per t1 increment, a relaxation delay of 1.0 s, SW of 10 563.69 Hz along t1 and 4795.40 Hz along t2, 1024 x 256 complex points in t2 and t1, respectively. In this case, the ^1^H‐^13^C HSQC constant time version ([^1^H‐^13^C] CT HSQC) was used with a heteronuclear coupling constant JXH = 145 Hz, constant time of 26.6 ms. The ^1^H‐^13^C CT HSQC was apodized with a square cosine window function and zero filled to a matrix of size 2048 x 1024 before Fourier transform and baseline correction. Aromatic 2D hetero‐nuclear [^1^H‐^13^C] HSQC spectrum was acquired with 128 scans per t1 increment, a relaxation delay of 1 s, SW of 6036.84 Hz along t1 and 4795.40 Hz along t2, 1024 x 200 complex points in t2 and t1, respectively. The aromatic [^1^H‐^13^C] HSQC spectrum was acquired with a JXH of 160 Hz, and the carrier frequency was set at 120 ppm for carbon. Successively, the spectrum was apodized with a square cosine window function and zero filled to a matrix of size 2048 x 2048 before Fourier transform and baseline correction.

^1^H, ^15^N, and ^13^C chemical shifts were calibrated indirectly by external DSS (4,4‐dimethyl‐4‐silapentane‐1‐sulfonic acid) reference and by using the internal DMSO‐d6. NMR spectra were processed by NMRpipe [39] and visualized using SPARKY [40] and CARA [41] software. NMR interaction studies of the melatonin with MT1 receptor were performed using isolated membranes of HEK293T cells overexpressing MT1‐receptor [24]. The NMR samples were prepared dissolving MT1‐membranes in 200 µL of PBS pH 7.8, 10% ^2^H_2_O and 10% DMSO‐d6; then, MLT was added in 2.0 fold molar excess.

1D and 2D ^1^H NMR experiments were acquired on MLT in the presence of cell membranes overexpressing MT1 receptor. As control, we acquired the same series of 1D and 2D ^1^H experiments on a sample containing the same concentration of MLT and membranes lacking the MT1 receptors. In details, 1D ^1^H experiments were acquired with SW of 7211.54 Hz, relaxation delay 1.0, 128 K data points for acquisition and 16 K for transformation. 2D [^1^H‐^1^H] TOCSY spectra were measured using the following acquisition parameters: 32 scans for t1 increment, SW of 8403.36 Hz along both t1 and t2, a relaxation delay of 1.0 s and 2048 x 200 complex points in t2 and t1, respectively. 2D [^1^H‐^1^H] TOCSY experiments were apodized with a square cosine window function and zero filled to a final matrix of 4096 x 4096 before the Fourier transform and baseline correction.

The relative AFs for individual protons of MLT were calculated using the following equation

$$\mathrm{AF}(\%)=(1‐I/I_{0})\;\times \;100\%$$

in which *I* is the individual peak intensity of each well‐resolved resonance of MLT obtained from the NMR spectra acquired in the presence of membrane overexpressing MT1 receptors; whereas *I*_0_ is the ^1^H signal intensity obtained for the same resonance in the ^1^H 1D spectrum of MLT acquired in the presence of membrane lacking the receptors (control experiment).

### 4.5 Homology Modeling Molecular Docking Studies

3D structural models of MT1 receptor in the active and inactive states were obtained using homology modeling methodologies by Modeller software [26]. In particular, the 3D structural model of the inactive MT1 receptor was built using the crystal structure of MT1 bound to ramelteon (PDB ID code: 6ME2) [21]; whereas for the active state of the MT1 receptor the 3D structure was calculated using as template: i) the cryo‐EM structure(PDB ID: 7DB6 [23] of the human MT1‐Gi signaling complex for the TM bundle (TM1–TM7) region comprising the residues 22–306; ii) the crystal structure of the human adenosine A2A receptor (PDB ID: 2YDO) [42] to better define the region from 291 to 308 comprising ICL4 and helix 8. In both cases, the disulphide bridge between the Cys100 (TM3) and Cys177 (ECL2) was explicitly defined during the molecular modeling. For both inactive and active MT1 models, a total of 300 conformations were calculated and were scored, after MD and simulating annealing optimization, by using the discrete optimized protein energy potential. After that, for each MT1 state the 3D model having the lowest score was selected as reference structure and the quality of the predicted structural model was evaluated by inspection of the Ramachandran Plot obtained using PROCHECK‐NMR [43]. To predict the 3D structural model of MLT in complex with the active MT1 receptor (*active*‐MT1/MLT complex) molecular docking calculations were performed using AutoDock Vina [27]. In the specific case, the molecular docking studies involved the following steps: i) preparing the starting coordinate files for MLT and the active MT1 receptor to include in the calculation structural information as spatial charges, polar hydrogen atoms, atom types and torsional degree of freedom; ii) AutoGrid routine: the molecular docking calculation requires predefined grid maps that are defined by the tool AutoGrid. The 3D lattice of the grid map was centered on a specific region of the MT1 receptor based on structural data reported for other GPCR‐agonist complexes [21, 23] and the grid size was set to be 30 x 30 x 40 and the grid spacing was 0.375 Å; iii) Docking procedure through AutoDock routine: a docking file, containing all input parameters for the docking calculation, was created using the AutoDock tools. The Lamarckian Generic Algorithm as searching method was used, minimized MLT molecule was randomly positioned into the grid box and the calculation was performed with 10 torsional degrees of freedom, number of energy evaluations = 25 000 000 and run number = 100 conformers. These latter were clustered using a RMSD cutoff of 2 Å. Finally, the 3D structural model of the most populated cluster having the lowest energy binding and well supported by ^1^H NMR‐based structural data was selected as reference structure. The structure of the *active*‐MT1/MLT receptor, as well as that of the *inactive*‐unbound MT1, was visualized and analyzed by PyMoL [44] and UCSF Chimera [45] software.

### 4.6 Molecular Dynamics Simulations

Molecular simulations were performed to get insights into the structural dynamics of the human melatonin receptor (uniprot id P48039) in both the *inactive*‐unbound state and upon MLT binding (MLT/MT1 complex). MLT parametrization was obtained using the Antechamber suite with the AM1‐BCC method and subsequently inspected and processed by the LEaP module [46]. The MD simulation package Amber v20 [47] was used to perform computer simulations by applying the Amber‐ff14SB [48] force field. The structures were centered in triclinic simulation boxes at 12 Å from each box edge and solvated with explicit water molecules (TIP3P model [49]), counterions were added to neutralize the system. After minimizations, systems were subjected to an equilibration phase where water molecules and protein heavy atoms were position restrained, and then, unrestrained systems were simulated in an NPT ensemble using the Langevin equilibration scheme to keep temperature and pressure constant (300 K, 1 atm). Electrostatic energies were evaluated by the particle mesh Ewald method [50] and Lennard–Jones forces by a cut‐off of 10 Å. All bonds involving hydrogen atoms were constrained using the SHAKE algorithm [51]. Periodic boundary conditions were imposed in all three dimensions and the time step was set to 2 fs. To enhance sampling two independent replicas were run for the two systems with different initial velocities (200 ns × 2 × system = 0.8 µs). Membrane systems were built using the membrane building tool CHARMMGUI [52, 53]. All the simulated systems were assigned for Amber‐ff14 SB force field and MLT ligand parametrization assigned by AM1‐BCC method, in line with MT1 simulations in water. MT1 in its unbound state and in complex with the MLT were embedded in 128 POPC lipids, coupled with TIP3P water molecules and 0.15 M NaCl. The final systems contained ∼105 000 atoms, with a volume of rough 100 × 100 × 113 Å3. Minimization and equilibration steps (1,5 ns) were run at decreasing restraints prior to MD production (2 replicas for 200 ns per system).

MD‐derived routine analyses (RMSD, RMSF, Gyration Radius, DSSP) were performed using the AmberTools package [54]. Cluster analysis was carried out on the concatenated 0.4 µs trajectory from the two replicas per system, selecting the hierarchical agglomerative (bottom‐up) approach and the average‐linkage algorithm to calculate distance between members of two clusters (distance cutoff = 3). Definitions and measurements of TMs rotational axes were performed by UCSF Chimera package [45], as reported elsewhere [55].

PCA has been applied to MD simulations to extract functionally relevant movements [56]. The largest collective fluctuations that account for the largest conformational variation were recovered by the principal eigenvectors (essential modes) of the covariance matrix of the given dynamic ensemble. PCA analysis was carried out on Cα atoms of the MT1 protein.

MM/PBSA calculations. Binding free energies (ΔG) were estimated using the MM/PBSA approach implemented in MMPBSA.py, employing equilibrated snapshots of the MD trajectories of the MT1‐MLT complex in different replicas [54]. Within the same framework, alanine scanning was performed to evaluate the contribution of key residues, yielding ΔΔG values.

## Supporting Information

Additional supporting information can be found online in the Supporting Information section.

## Author Contributions

Luigi Russo conceived and supervised this study. Francesca Scebba e Debora Angeloni prepared cell membranes. Clementina Acconcia and Luigi Russo acquired and analyzed NMR data. Clementina Acconcia carried out molecular docking calculations. Antonella Paladino performed Molecular Dynamics simulations. Luigi Russo and Stefano Comai and drafted the manuscript. All authors contributed to the analysis and interpretation of the data, as well as the preparation of the manuscript. All authors read and approved the final version of the manuscript.

## Funding

This study was supported by PRIN PNRR 2022 (P2022YBTKF), CINECA (HP10CBDF0C).

## Conflicts of Interest

The authors declare no conflicts of interest.

## Supporting information

Supplementary Material
