# Supplementary material for: Structural and Dynamics Insights into Melatonin Binding to MT1 Receptor
Source: Chembiochem. 2026 May 15;27(10):e70376. doi: 10.1002/cbic.70376 (PMC13178210; doi:10.1002/cbic.70376)
Supplement: Supplementary file 1 — Supplementary Material [file CBIC-27-e70376-s001.pdf]

Supporting Information
For

Structural and dynamics insights into melatonin binding to MT1 receptor.

Clementina Acconcia[1], Antonella Paladino[2], Francesca Scebba[3], Maria della Valle[1,4], Martina Montebuglio[2], Gaetano Malgieri[1], Carla Isernia[1], Roberto Fattorusso[1], Debora Angeloni[3,5], Stefano Comai[6,7,8,9], Luigi Russo[1*].

[1]Department of Environmental, Biological and Pharmaceutical Sciences and Technologies, University of Campania "L. Vanvitelli", Caserta, Italy.
[2]Institute of Biostructures and Bioimaging, CNR, Via Castellino 111, 80131 Naples, Italy.
[3]Health Science Interdisciplinary Center, Scuola Superiore Sant'Anna, Via G. Moruzzi, 56124 Pisa, Italy.
[4]Institute of Crystallography, CNR, Via Vivaldi, 43, 81100, Caserta, Italy.
[5]The Institute of Biorobotics, Scuola Superiore Sant'Anna, Via G. Moruzzi, 56124 Pisa, Italy.
[6]Department of Pharmaceutical and Pharmacological Sciences, University of Padua, Padua, Italy.
[7]Department of Biomedical Sciences, University of Padua, Padua, Italy.
[8]Department of Psychiatry, McGill University, Montreal, QC, Canada.
[9]IRCCS San Raffaele Scientific Institute, Milan, Italy.

Table of Contents

Figure S1. Flow-chart illustrating the integrated approach used for studying the MT1-MLT interaction.

Figure S2. ^{1}H, ^{15}N and ^{13}C chemical shifts assignment of the free melatonin.

Figure S3. NMR experiments of MLT acquired in absence in presence of membranes with and without MT1 receptors

Figure S4. NMR binding studies of MLT to MT1 receptor using membrane preparation.

Figure S5. Structural quality assessment.

Figure S6. Structural analysis of the MD results of the *inactive*-unbound MT1.

Figure S7. Cryo-EM structure of the *active*-MT1 bound to ramelteon.

Figure S8. Ligand binding modes of MT1 receptor in the *inactive* and *active* forms.

Figure S9. Comparison of the three-dimensional models obtained by homology modeling for *inactive*-unbound MT1 and *active*-MT1/MLT complex.

Figure S10. Principal Component Analysis of the MT1 receptor

Figure S11. Structural superposition of the *inactive*-unbound MT1 and *active*-MT1/MLT systems.

Figure S12. Transmembrane Helix6 motions.

Table S1. Structural displacement of TM5 and TM6 helices along the simulation time.

Table S2. In silico alanine scanning by MM-PBSA calculations. ΔΔG values obtained for the Q181A and Y281A mutants are averaged over two replicas.

Figure S1. Flow-chart illustrating the integrated approach used for studying the MT1-MLT interaction.

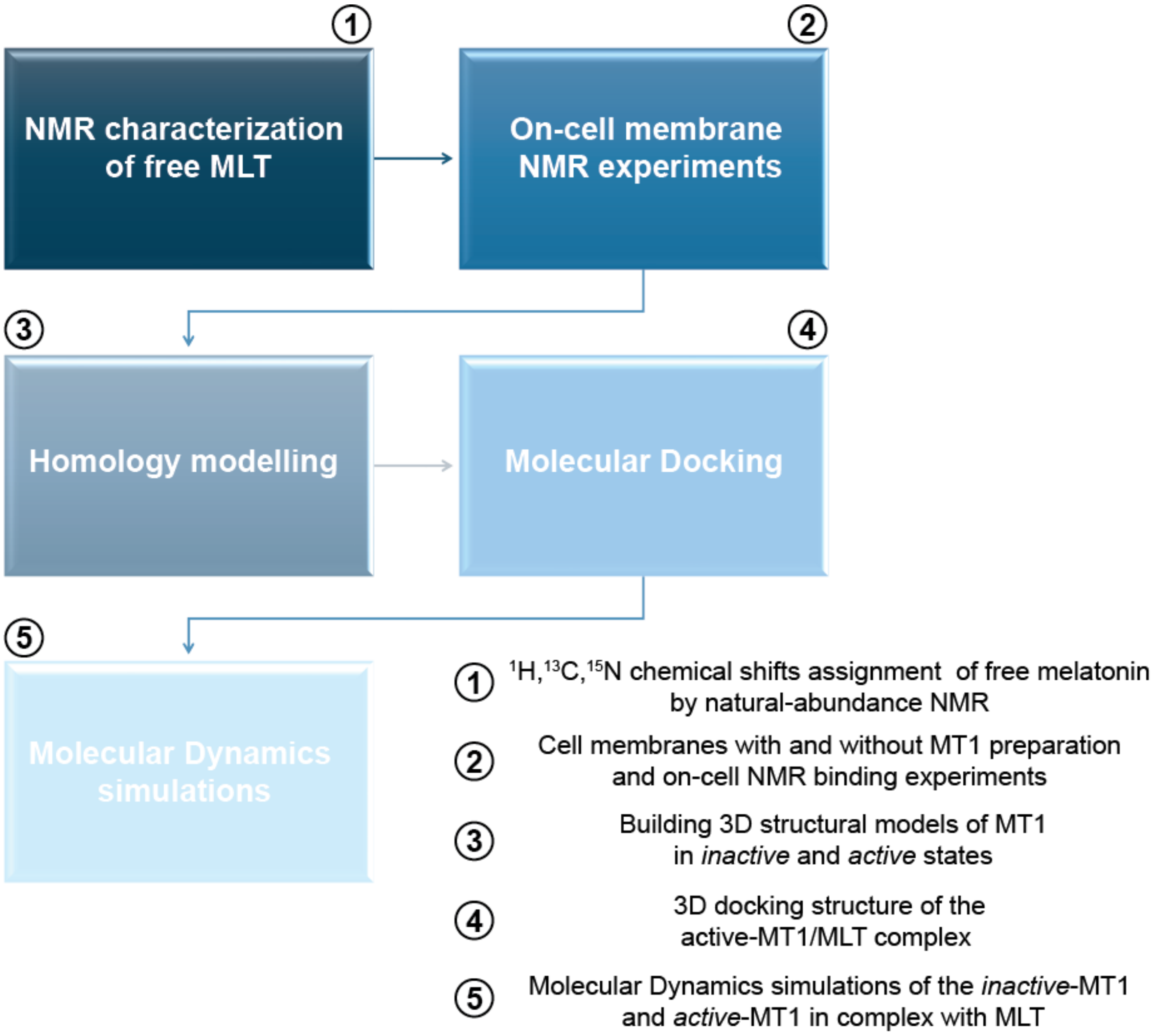

Figure S2. ^{1}**H,** ^{15}**N and** ^{13}**C chemical shifts assignment of the free melatonin.** The resonances were assigned using a 600 MHz spectrometer at 298K.

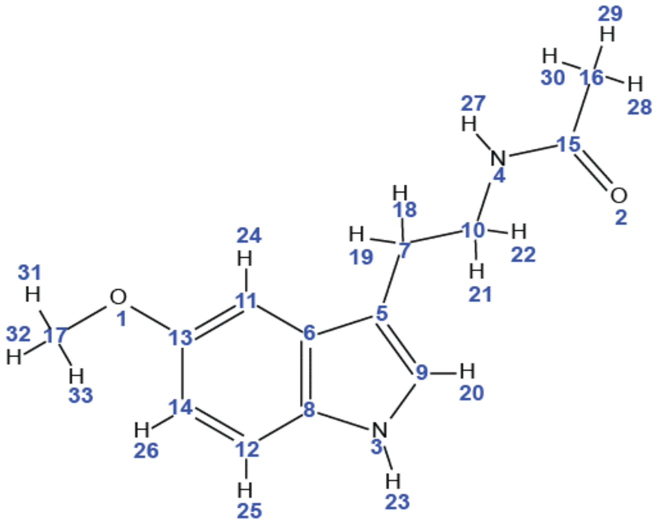

Atom	δ^{1}H (ppm)
H(18,19)	2.80 ± 0.01
H20	7.07 ± 0.01
H(21,22)	3.33 ± 0.01
H23	9.83 ± 0.01
H24	7.09 ± 0.01
H25	7.28 ± 0.01
H26	6.77 ± 0.01
H27	7.77 ± 0.01
H(28,29,30)	1.76 ± 0.01
H(31,32,33)	3.74 ± 0.01

Atom	δ^{13}C (ppm)
C7	26.92 ± 0.50
C9	103.49 ± 0.50
C10	42.85 ± 0.50
C11	126.90± 0.50
C12	115.47 ± 0.50
C14	114.26 ± 0.50
C16	24.80 ± 0.50
C17	58.72 ± 0.50

Atom	δ^{15}N (ppm)
N3	126.88 ± 0.50
N4	126.40 ± 0.50

Figure S3. NMR experiments of MLT acquired in absence and in presence of membranes with and without MT1 receptors. (A) A portion of the 2D [^{1}H-^{1}H] TOCSY spectrum of MLT acquired at 298K using a 600 MHz spectrometer. (B) Expansion region of 2D ^{1}H-^{1}H TOCSY spectra of MLT acquired in the presence of membranes without (light blue) and with (red) MT1 receptors. (C) Portion of synthetic ^{1}H NMR spectrum obtained by subtracting the spectrum measured as control to the ^{1}H spectrum of MLT with MT1-membranes.

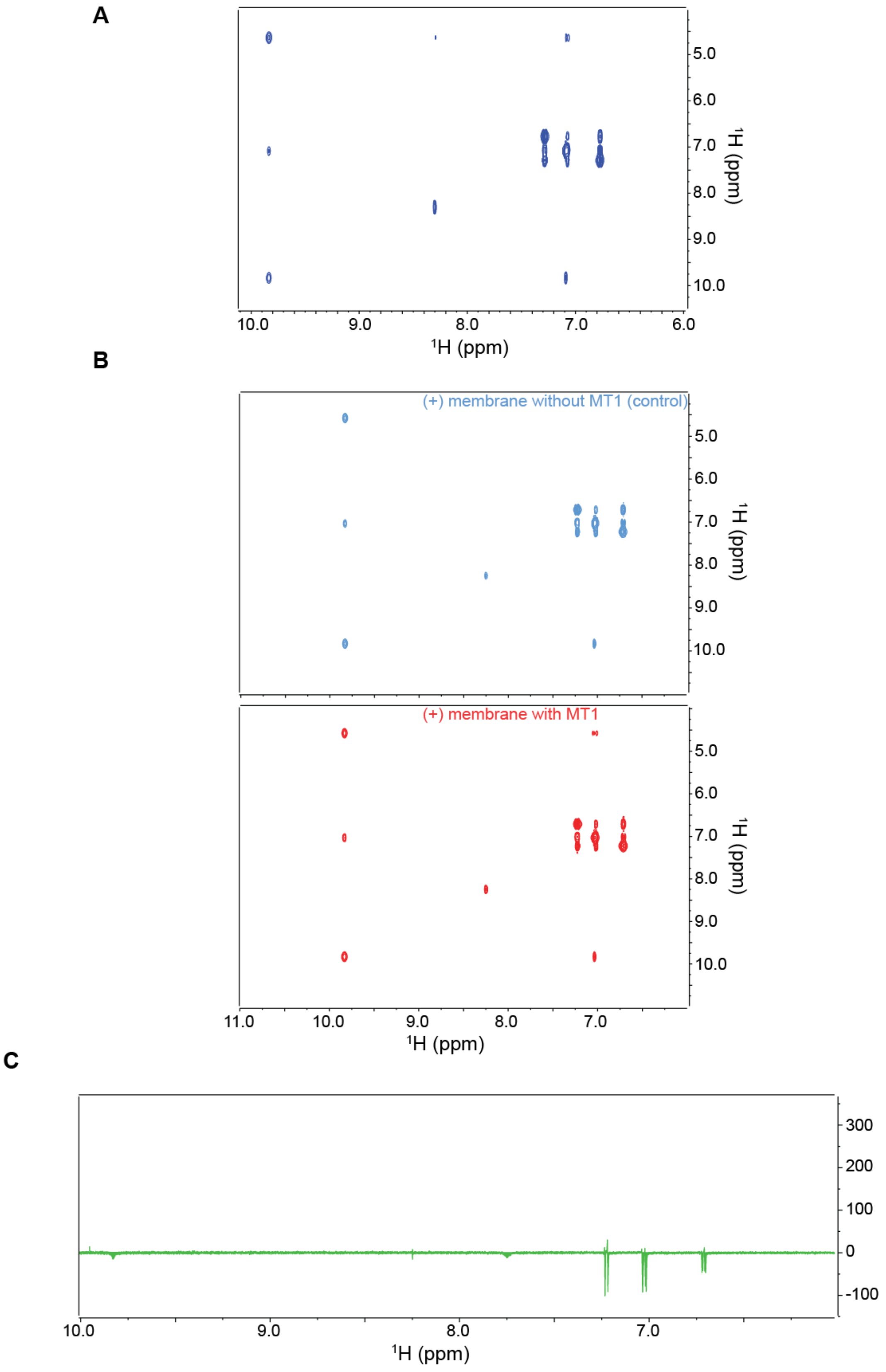

Figure S4. NMR binding studies of MLT to MT1 receptor using membrane preparation. Relative attenuation factors AFs (%) obtained, as reported in materials and methods, by comparing the individual signal intensities in the ^{1}H NMR spectra of MLT acquired upon addition of cell membranes with and without overexpressed MT1 receptor. The melatonin atoms are depicted by color code: yellow (< 10 % AF), orange (40 ≤ AF ≤ 50 %) and red (50 ≤ AF ≤ 60 %). For the protons of the acetyl CH3 (H(28), H(29) and H(30)) the smallest AF-% was observed suggesting that this portion of MLT is less involved in the MT1 binding; the HN proton (H27) of the amide group and protons of the methylene unit showed AF-% values ranging from 40 to 50 %; finally, similar AF-% values, ranging from 50 to 60 %, were observed for the protons located within the indole unit and the methoxy group.

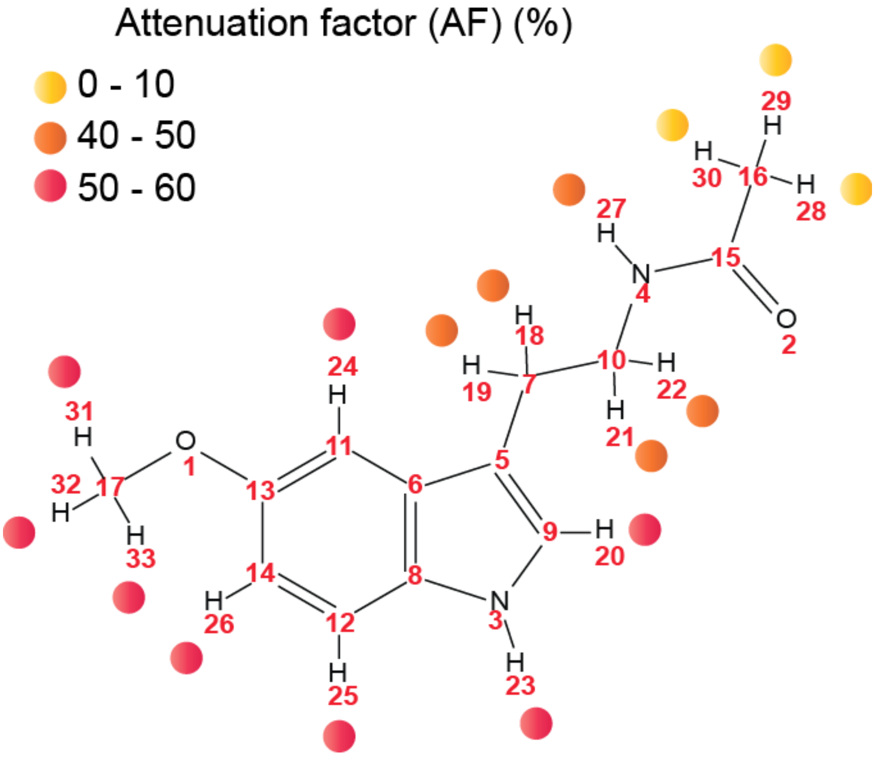

Figure S5. Structural quality assessment. Ramachandran plot analysis of the *inactive*-unbound MT1 (A) and *active*-MT1/MLN complex (B) structural models.

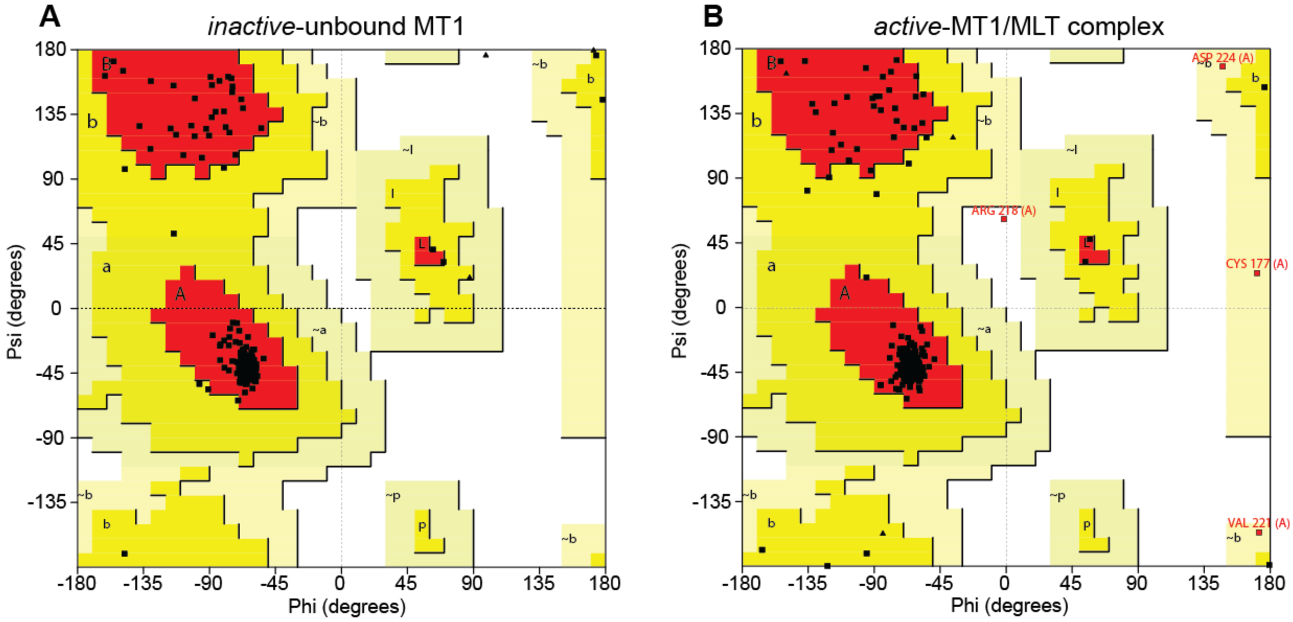

Residues in most favoured regions	96.3%
Residues in additional allowed regions	3.7%
Residues in generously allowed regions	0.0%
Residues in disallowed regions	0.0%

Residues in most favoured regions	95.2%
Residues in additional allowed regions	3.3%
Residues in generously allowed regions	1.1%
Residues in disallowed regions	0.4%

Figure S6. Structural analysis of the MD results of the *inactive*-unbound MT1. Time-dependent evolution of the secondary structures elements of the receptor during the two simulation runs.

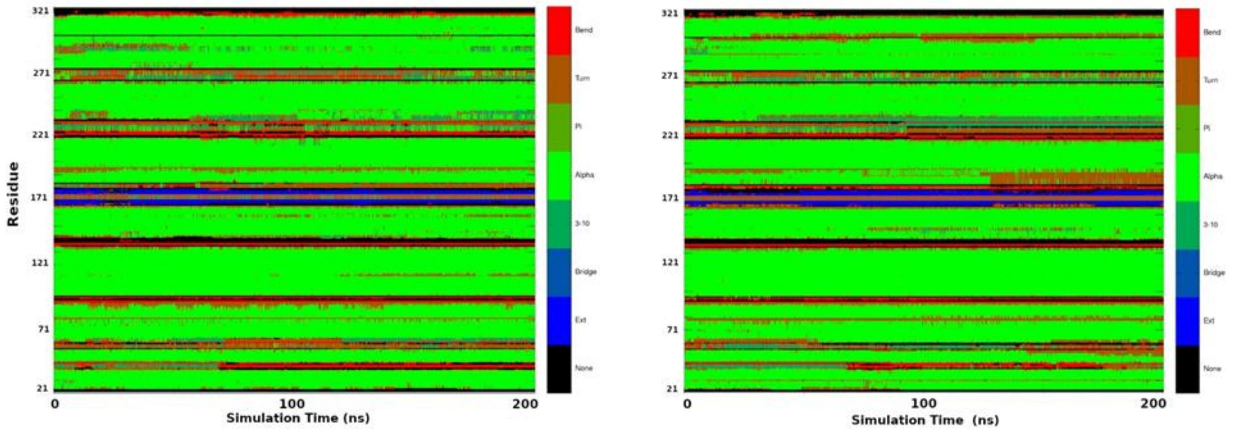

Figure S7. Cryo-EM structure of the *active*-MT1 bound to ramelteon. (A, B) Ribbon drawing representation of the *active*-MT1/ ramelteon complex in two orientations rotated of 90° around x axis. The active form of the MT1 receptor is shown in light grey ribbon drawing representation whereas the ligand is reported in dark cyan stick representation. (C) Comparison of the orthosteric binding pocket of the *active*-MT1in complex with ramelteon (dark cyan) and MLT (yellow). (D) Schematic representation of interactions between *active*-MT1 and ramelteon as reported by LigPlot+ software.

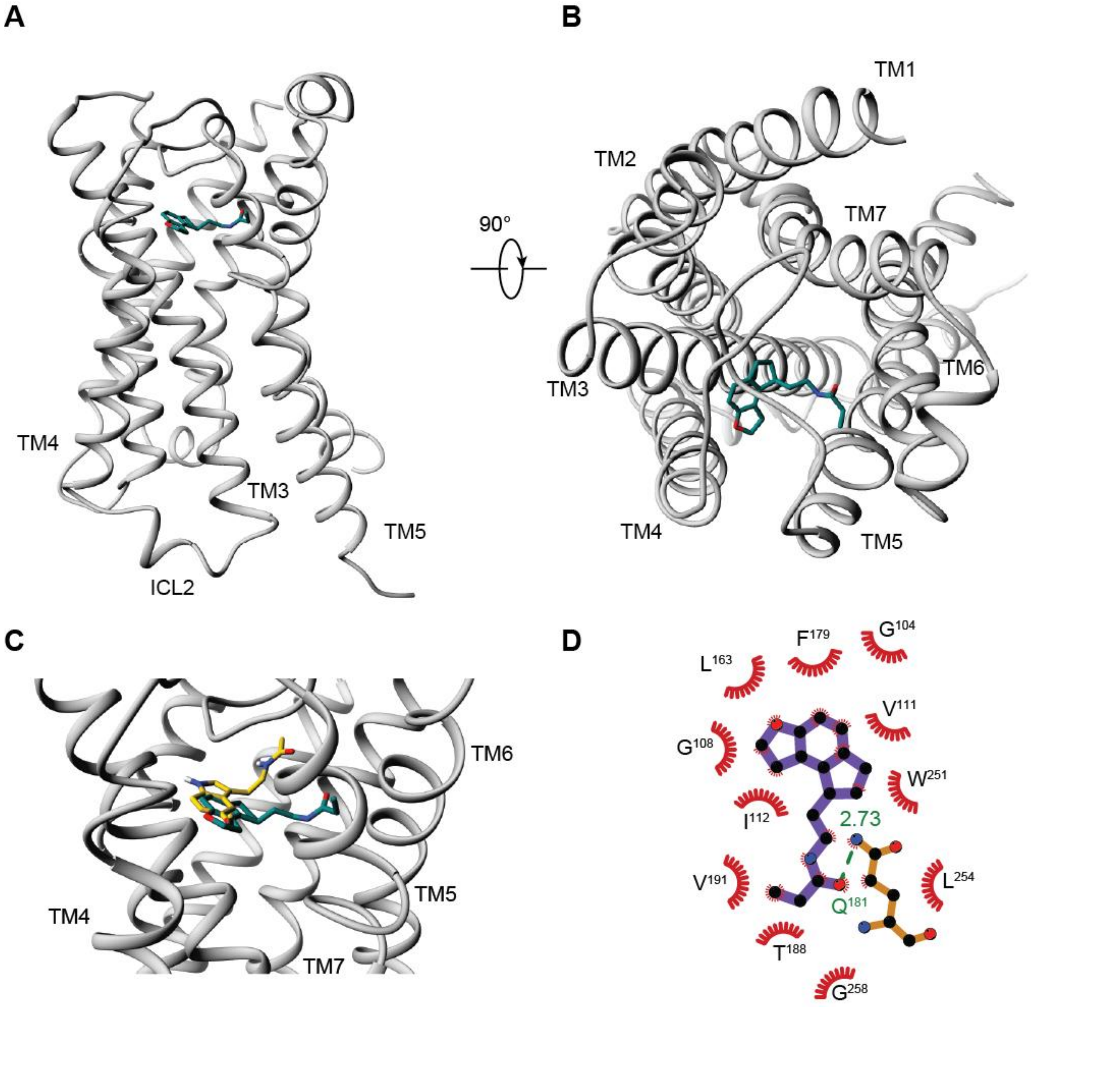

Figure S8. Ligand binding modes of MT1 receptor in the*inactive* and *active* forms. Comparison of the orthosteric binding pocket of *inactive*-MT1/agonist complexes (ramelteon (light blue) (A),2-phenylmelatonin (medium purple) (B) and 2-iodomelatonin (magenta) (C)) with *active*-MT1 bound to MLT (yellow). The MT1 receptor is shown in light grey ribbon drawing representation whereas the ligands are reported in stick representation. The schematic representation of interactions between *intactive*-MT1 and ramelteon (A),2-phenylmelatonin (B) and 2-iodomelatonin (C), are also reported as analyzed in LigPlot+ software.

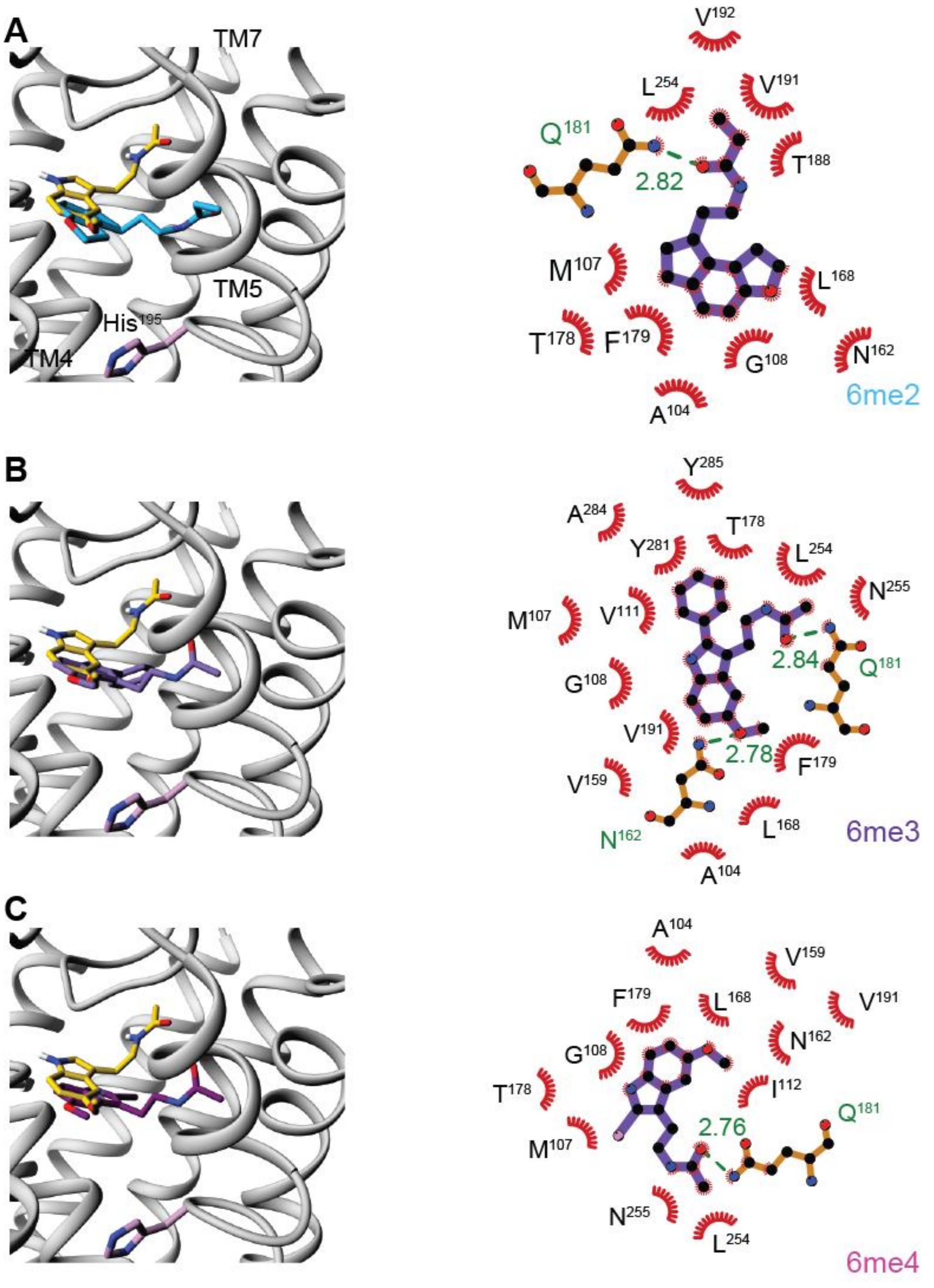

Figure S9. Comparison of the three-dimensional models obtained by homology modeling for *inactive*-unbound MT1 and *active*-MT1/MLT complex. Overlay of the protein structures of the *inactive*-unbound MT1 (light grey) and the *active*-MT1 (slate gray) bound to MLT (yellow) in n two orientations (A, B) rotated of 90° around x axis.

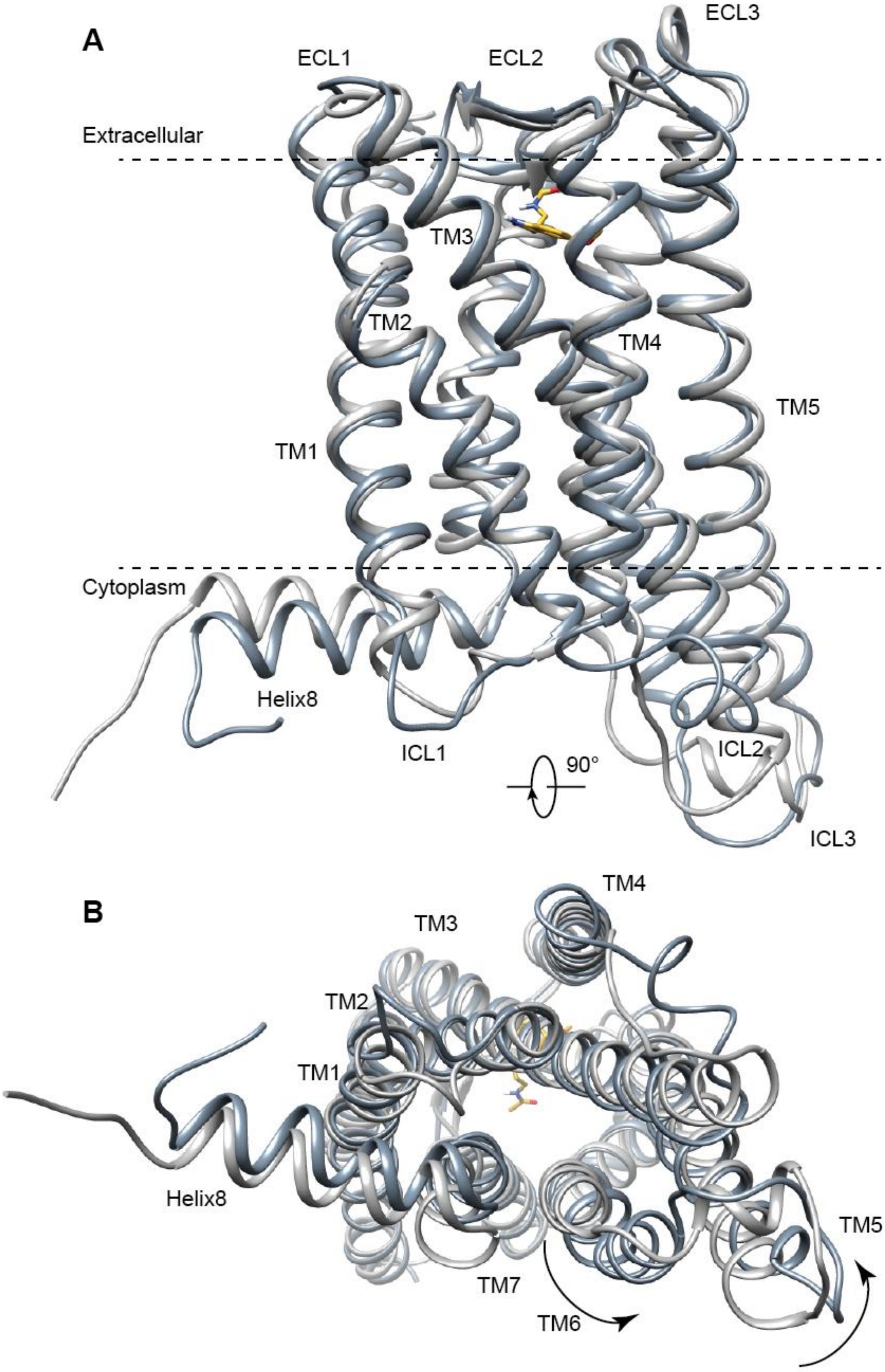

Figure S10. Principal Component Analysis of the MT1 receptor. The covariance matrix was built on Cα atoms: eigenvector 1 and 2 jointly collect about 53% and 61% of the total variance of the simulations of the free and bound-MT1 receptor, respectively. Extreme conformation projections along the principal modes of the unbound (A) and MT1-MLT complex (B) are shown as traces, colored red-to-blue from the N- to the C-terminus. The first two principal components (eigenvector 1 and 2) were used to project the MD trajectories onto the essential space explored by the bound system (C): black dots indicate the MT1-MLT complex, while red dots correspond to the unbound MT1 receptor.

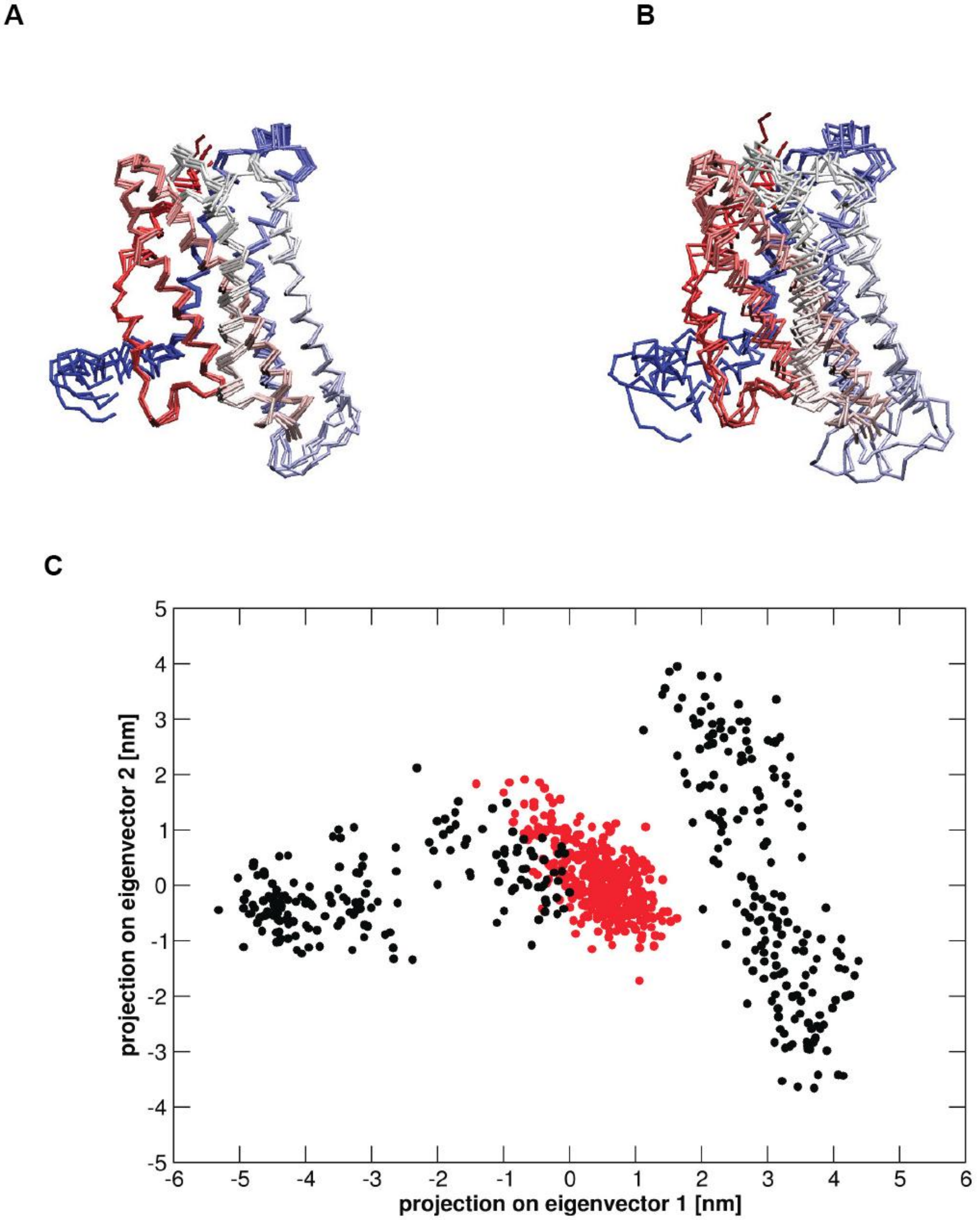

Figure S11. Structural superposition of the *inactive*-unbound MT1 and *active*-MT1/MLT systems. (A) Solid cartoons are used for inactive-unbound MT1 system (first cluster ~ 44%) and ghost cartoons for *active*-MT1/MLT complex (first cluster ~30%); (B) Zoom-in into the TM5-TM6 superposition between active-MT1/MLN complex (solid cartoons) and inactive-unbound MT1 (ghost cartoons).

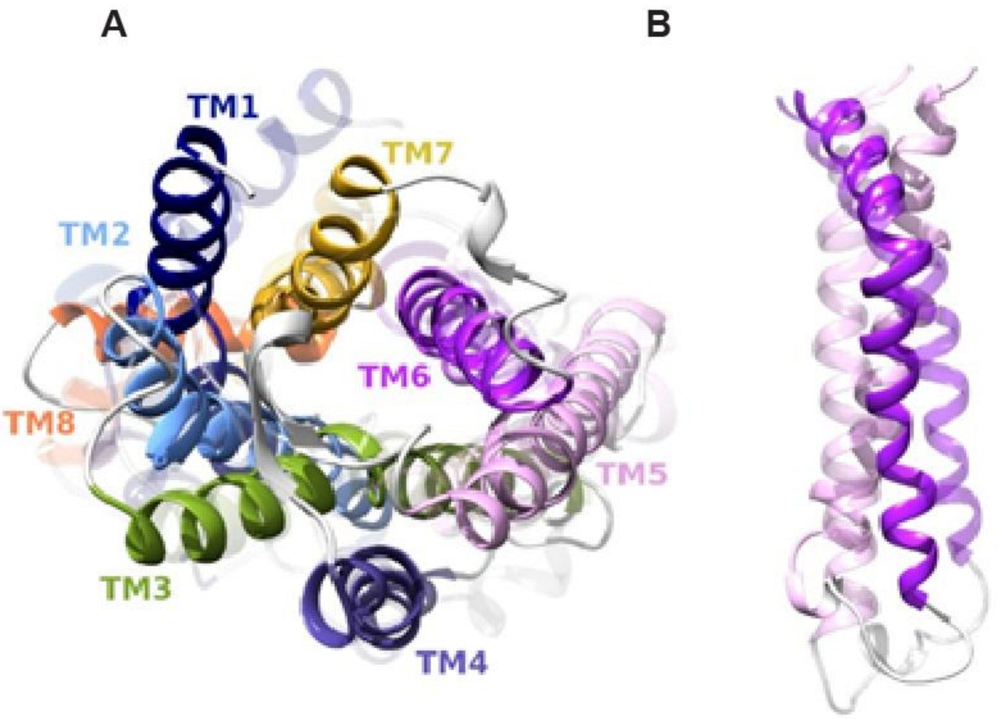

Figure S12. Transmembrane Helix6 motions. Inter-distances between pairs of transmembrane helices are plotted for the inactive-unbound MT1 (upper panel) and *active*-MT1/MLT (bottom panel) systems are plotted. Distributions of the distances between the geometric center of mass for each TM are calculated over the concatenated trajectory from the 2 replicas in solution.

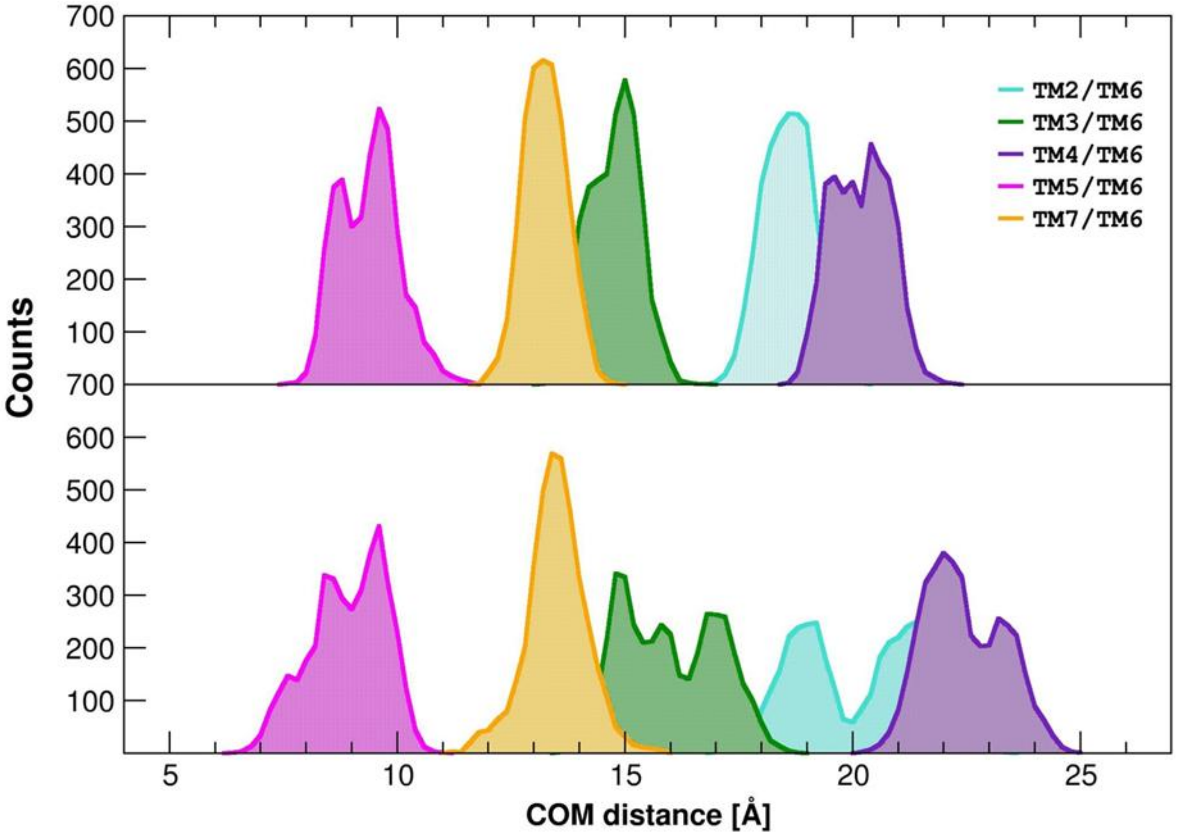

Table S1. Structural displacement of TM5 and TM6 helices along the simulation time.

	Cα RMSD (Å) ± Std.Dev.		
	**TM5**	**TM6**	**TM5/TM6**
inactive-unbound MT1	1.45 ± 0.40	1.38 ± 0.42	3.11 ± 0.35
active-MT1/MLT complex	2.23 ± 0.30	2.22 ± 0.49	4.22 ± 0.58

Table S2. *In silico* alanine scanning by MM-PBSA calculations. ΔΔG values obtained for the Q181A and Y281A mutants are averaged over two replicas.

	time 0 ns	***time 200 ns***
**MUTANT**	**<ΔΔG>** kcal/mol	**<ΔΔG>** kcal/mol
**Q181A**	-1.12	1.47
**Y281A**	-2.0	2.22
